# Research on recognition and classification of pulse signal features based on EPNCC

**DOI:** 10.1038/s41598-022-10808-6

**Published:** 2022-04-25

**Authors:** Haichu Chen, Chenglong Guo, Zhifeng Wang, Jianxiao Wang

**Affiliations:** School of Mechatronic Engineering and Automation, University of Foshan, Nanhai District, Foshan, Guangdong China

**Keywords:** Bioinformatics, Computational biology and bioinformatics, Classification and taxonomy

## Abstract

To rapidly obtain the complete characterization information of pulse signals and to verify the sensitivity and validity of pulse signals in the clinical diagnosis of related diseases. In this paper, an improved PNCC method is proposed as a supplementary feature to enable the complete characterization of pulse signals. In this paper, the wavelet scattering method is used to extract time-domain features from impulse signals, and EEMD-based improved PNCC (EPNCC) is used to extract frequency-domain features. The time–frequency features are mixed into a convolutional neural network for final classification and recognition. The data for this study were obtained from the MIT-BIH-mimic database, which was used to verify the effectiveness of the proposed method. The experimental analysis of three types of clinical symptom pulse signals showed an accuracy of 98.3% for pulse classification and recognition. The method is effective in complete pulse characterization and improves pulse classification accuracy under the processing of the three clinical pulse signals used in the paper.

## Introduction

Pulse signals often contain a wealth of important information, such as the human heart and cardiovascular system. Pulse wave pressure and pulse waveform characteristics are important bases for evaluating the physiological and pathological status of the human cardiovascular system \* MERGEFORMAT^1^. Both the pulse diagnosis in Chinese medicine and the examination of cardiovascular diseases in Western medicine attempt to extract various physiological and pathological information from the pulse wave features. However, due to the complexity and diversity of the pulse signal, enabling a complete characterization of the pulse signal has become one of the problems that many scholars need to solve.

To improve life expectancy and reduce healthcare costs, PPG has emerged as a promising technique for early screening^2^. It is of increasing interest to researchers because of its noninvasive, inexpensive, and convenient diagnostic features. The signal acquired by PPG is pulsatile, identifies relevant features of blood flow activity, and can be used to measure cardiac output^3,4^. PPG has been widely accepted by the International Organization for Standardization (ISO) and the European Committee for Oxygen Saturation Measurement. When a finger is placed on the PPG device, it can easily record a high-quality pulse signal^5–8^. Pulse signals from PPG were preprocessed to improve signal quality and estimate heart rate^9–11^.

In the area of pulse signal feature processing, many scholars have conducted a series of researches on the extraction and classification of pulse features. For example, Natalia^12^ proposed the use of wrist pulse wave signals to analyze hemodynamic problems in hypertensive patients. The results showed that establishing pulse image analysis (PIA) in hypertensive patients can help in the early identification of target organ damage (TOD) and uniform pulse diagnosis. Hui Liu^13^ proposed to calculate blood flow parameters based on the characteristic quantity K of the pulse waveform and analyzed the correlation between K values and blood flow parameters. The results show that the method is simple and fast, and can use the pulse wave area characteristic quantity can accurately predict the changes of blood flow parameters. Wang^14^ developed a mechanical model of the pulse wave system, in which the pulse wave system is considered as a system of vital energy that propagates in the blood in the form of waves. Hsing-Chung Chen^15^ used an image fitting method to segment the arterial pulse wave curve, extracted its slope and period as pulse time-domain features, and compared them with the difference of the mean feature matrix. Finally, the optimal pulse period curve for physicians' reference analysis was obtained. Hadiyoso^16^ used the decomposition method of EEMD for the extraction of RR breathing waves. By comparing the performance of EMD and VMD, the study concluded that EEMD has better performance.

Lorenzo^17^ used machine learning (ML) and deep learning (DL) to study the feasibility of the pulse signal obtained via PPG and to predict vascular aging. The results show that ML is more useful to analyze the underlying biological properties of HVA predictions when DL is computationally inexpensive. Nogueira^18^ computed the features of each segment of the heart sound signal by extracting a set of time-domain features and two frequency domain features and classified the heart sound signal using radial basis algorithm and finally obtained a classification accuracy of about 83.22%. Rubin^19^ classified the MFCC heat map by segmenting the heart sound signal and described in detail the generated neural network structure and design decisions. The MFCC method was used to transform the frequency domain features, but in the presence of external interference factors, the performance of MFCC is reduced, which affects the accuracy of the study results. Hu^20^ proposed a classification method based on the Shannon energy envelope, Hilbert transform, and deep convolutional neural network. The results showed that it significantly outperformed other commonly used methods when the features were unclear. Zhang^21^ proposed to classify about 2280 PPG signals using a 9-layer one-dimensional convolutional neural network and investigated its correlation with atherosclerosis, and finally obtained a classification accuracy of 93%.

Choon-Hian Gohacf^22^ proposed to extract the features of PPG signals using CNN and normalize the features using the z-score normalization method. Finally, the overall accuracy of this network model is 94.5%. Hu^23^ proposed to obtain pulse features by continuous wavelet transform and finally processed by the convolutional neural network to obtain 83.81% classification accuracy. Reit Kavsao Lu^24^ extracted time-domain features by first and second-order derivatives of the PPG signal and ranked the contribution of biometric features based on these features. Finally, a k-NN classifier was used for validation and an average accuracy of about 90.7% was obtained. Tripti^25^ proposed the classification of samples by mean clustering strategy and fine Gaussian multiclass support vector machine. PPG signals from patients with various diseases were investigated and the final results showed its applicability in identifying unique heart-related diseases. In addition to screening for diseases, PPG signals have also been applied for blood pressure prediction^26–28^. Sun^29^ proposed the use of HHT-based convolutional neural networks for blood pressure detection and classification of PPG signals. The accuracy of classification was 98.9% for normotension, 85.8% for hypertension, and 93.54% for prehypertension. Feature extraction is performed on traditional methods. For example, feature statistics and screening based on PPG signal features^30–33^. Ramachandran^34^ proposed to use features such as singular value decomposition, statistical features, and wavelets and then applied softmax and Gaussian mixture model classifier to classify various risk levels of cardiovascular diseases. Finally, the classification accuracy obtained was 97.88%. Poulomi^35^ analyzed time-domain features and then classified patients and healthy subjects using decision trees, discriminant analysis, logistic regression, support vector machines, KNN, and augmented trees. Finally, a classification accuracy of 94% was obtained.

To make a complete characterization of the pulse signal for achieving pulse classification recognition, the EPNCC-based method is used in the paper to obtain the frequency domain features and the wavelet scattering method is used to obtain the time-domain features. The time–frequency features are fused and input to a convolutional neural network for recognition. The proposed method is validated experimentally on the MIT-BIH-mimic database. We wish to provide new methods and ideas for the application of pulse signals in clinical diagnosis.

## PPG feature processing method

### PPG temporal feature extraction

**Figure 1:**
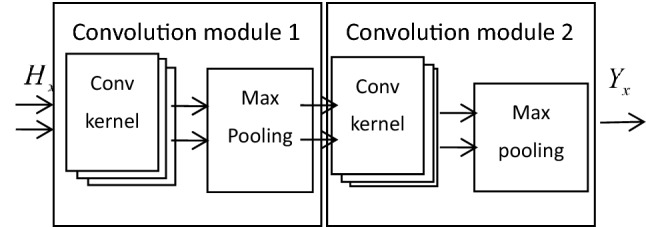
PPG temporal feature extraction network.

The pulse long-time signal contains the internal characteristics of each cycle and the characteristic relationships between the cycles. It reflects the envelope variation of the pulse signal and is the time-varying characteristic of the pulse signal. It still has a classification effect for pulse signals when the body is in a state of motion change. Multi-period signal data has higher dimensionality than single-period signals and can contain more feature information. Therefore, in this paper, a multi-period pulse signal is selected as the data sample. More importantly, using an appropriate convolution module, it is possible to reduce the data dimensionality while still retaining the features that are not lost. The pulse period feature extraction process is shown in Fig. 1.

To facilitate the input of the convolution kernel for calculation, this article lists the corresponding pulse cycle signal as a matrix form of multiple cycles juxtaposed, satisfying:

1 $$H_{x} = [h_{1} (1),h_{1} (2), \ldots ,h_{1} (i);h_{2} (1),h_{2} (2),h_{2} (i); \ldots h_{M} (1),h_{M} (2),h_{M} (i)]^{{\text{T}}}$$

where M is the number of cycles.

When choosing the actual number of M, a suitable value needs to be selected because when the selected value of M is too small, the variation of the pulse period cannot be obtained. However, when the value of M is chosen too large, the number of dimensions of the network input increases, which will increase the complexity of the network computation. Therefore, in this paper, the M value of the pulse period sample data is chosen to be 5. To obtain good results while reducing the computational cost, this paper first uses two convolution modules to extract the temporal characteristics of the pulse. Each convolutional module contains a convolutional layer, an activation layer, and a pooling layer.

Each time the input is convolved, the convolution kernel will output a feature mapping. To prevent gradient explosion and gradient message update of multilayer network coefficients, the ReLU function is chosen as the activation function of the convolution layer in this paper. The expression is: f(x) = max(0, x). ReLU, as an activation function, can improve the training speed of the network due to its linear and non-saturated form. After the activation function processing, the maximum pooling layer is selected as the final output of each convolutional module in this paper, which further reduces the size of the data.

After experimental verification, the parameter configuration of the convolution module used in this article is shown in Table 1. The convolutional network structure is shown in Fig. 1.

**Table 1:** Convolution module parameter configuration.

Conv module 1	Parameter	Conv module 2	Parameter
Dimension	5*1	Dimension	3*1
Conv step size	2	Conv step size	1
Number	32	Number	64
Activation function	ReLU	Activation function	ReLU
Method	Max pooling	Method	Max pooling
Pooling dimension	3*1	Pooling Dimension	3*1
Step size	2	Step size	1

### PPG signal wavelet scattering processing

A wavelet scattering method was born by combining the concepts of wavelet theory and signal processing. It introduces a depth feature map in the time series to make the local time of the series invariant and stabilizes the time distortion by a cascade of signal filtering and modular operators. The expression for wavelet scattering is^37^.

2 $$X[\alpha_{1} ,\alpha_{2} ]r = ||r*\psi_{{\alpha_{1} }} (x)|*\psi_{{\alpha_{2} }} (x)|*\phi_{j} ,\alpha_{1} < \alpha_{2} < j$$

Among them, ${\text{X}}\left[ {{\upalpha }_{1} ,{\upalpha }_{2} } \right]{\text{r}}$ represents the further decomposition of the wavelet scattering transform scale,$\left| {{\text{r}}*\uppsi _{{{\upalpha }_{1} }} \left( {\text{x}} \right)} \right|$ represents the wavelet transform modulus formula, and j represents the scale. Each feature averaging will cause the loss of high-frequency information, so it is necessary to continuously iterate the last wavelet scattering coefficient to ensure the conservation of energy. Finally, the scattering characteristic coefficients of the wavelet are:

3 $$X\left[ {\alpha_{1} ,\alpha_{2} \cdots \alpha_{n} } \right]r = |||r*\psi_{{\alpha_{1} }} (x)|*\psi_{{\alpha_{2} }} (x)| \cdots *\psi_{{\alpha_{n} }} (x)|*\phi_{j} ,\alpha_{1} < \alpha_{2} < \cdots \alpha_{n} < j$$

In this paper, a wavelet scattering network (WS) is used to preprocess the time domain features of pulse signals. This method can well characterize the band characteristics of its low and high-frequency signals. The expression of the output characteristic coefficients of the wavelet scattering network is

4 $$X_{j} [\alpha_{1} \ldots \alpha_{n} ]r = \left( \begin{gathered} X_{j} [\phi ]r \hfill \\ X_{j} [\Lambda_{j}^{1} ]r \hfill \\ X_{j} [\Lambda_{j}^{2} ]r \hfill \\ X_{j} [\Lambda_{j}^{3} ]r \hfill \\ \ldots \hfill \\ \end{gathered} \right) = \left( \begin{gathered} r*\phi_{{2^{j} }} \hfill \\ |r*\psi_{{\alpha_{1} }} |*\phi_{{2^{j} }} \hfill \\ ||r*\psi_{{\alpha_{1} }} |*\psi_{{\alpha_{2} }} |*\phi_{{2^{j} }} \hfill \\ |||r*\psi_{{\alpha_{1} }} |*\psi_{{\alpha_{2} }} |*\psi_{{\alpha_{3} }} |*\phi_{{2^{j} }} \hfill \\ \ldots \hfill \\ \end{gathered} \right)$$

The pulse period signal is processed by wavelet scattering (WS). After the parameters of the wavelet scattering decomposition frame are configured, the decomposition frame coefficients are expressed as SF, and then the ${\text{X}}_{{\text{j}}} \left[ {{\upalpha }_{1} ...{\upalpha }_{{\text{n}}} } \right]{\text{r }}$ scattering feature is obtained through the scattering feature function $\left[ {{\text{SF}},{\text{H}}_{{\text{X}}} } \right]$ as the input of the convolutional neural network. The SF parameter configuration is shown in Table 2, and the convolutional network structure is shown in Table 1.

**Table 2:** Wavelet scattering decomposition frame parameters.

Signal length (Number of points)	450
Invariance scale	1.8
Quality factors	[8 1]
Boundary	"Periodic"
Sampling frequency	125
Precision	"Double"
Over sampling factor	0

### Frequency-domain feature extraction of EPNCC

In the traditional PNCC method, the FFT is used for signal preprocessing, but the FFT does not work well for non-smooth signals. Therefore, we use EEMD to preprocess the signal instead of FFT to improve the PNCC method.

The EEMD method is used to preprocess the PPG signal samples. the steps of the EEMD algorithm are as follows: (1) add normally distributed white noise to the original signal; (2) add the white noise to the signal as a whole and then perform EMD decomposition to obtain each IMF component; (3) repeat steps 1 and 2, adding a new normally distributed white noise sequence each time; (4) integrate and average the IMF obtained each time as the final result are integrated and averaged as the final result.

After decomposing the pulse signal by EEMD, each IMF component and a residual are obtained. Due to the time-varying and random nature of the noise, the IMF components after EEMD decomposition will vary. The large differences in these IMF components are meaningless for characterizing the impulse signal properties. Therefore, before extracting the PNCC frequency domain features, a correlation analysis is performed on the EEMD decomposed IMFS components to filter out the components that can characterize the impulse signal. The correlation analysis can determine the coherence between each IMF component and the original PPG signal, and the expression formula for the coherence coefficient is:

5 $$\mu = \frac{{\sum\nolimits_{i = 1}^{M} {f_{k} (i)s_{k} (i)} }}{{\sqrt {\sum\nolimits_{i = 1}^{M} {f_{k}^{2} (i)\sum\nolimits_{i = 1}^{M} {s_{k}^{2} (i)} } } }}$$

Among them, ${\text{f}}_{{\text{k}}} \left( {\text{i}} \right)$ is i-th component of IMF, ${\text{s}}_{{\text{k}}} \left( {\text{i}} \right)$ is the difference between the original PPG signal and ${\text{f}}_{{\text{x}}} \left( {\text{i}} \right)$. Coherent IMFS is screened by setting the coefficient threshold.

After the PPG signal is processed by EEMD, the components are arranged and expressed by a matrix ${\text{IMF}}_{{\text{n}}} = \left[ {{\text{IMF}}_{1} ;{\text{IMF}}_{2} \ldots {\text{IMF}}_{{\text{n}}} } \right]$. EPNCC feature extraction steps:

1. Estimate the power spectrum of each component in ${\text{IMF}}_{{\text{n}}}$. Power spectrum calculation expression:
  6 $$P_{n} (\omega ) = \mathop {\lim }\limits_{T \to \infty } \frac{{|IMF_{n} |}}{2\pi T}$$
2. The power estimation is input to the Gammatone filter for filtering processing. The time-domain impulse response formula of the Gammatone filter is as follows:
  7 $$g\left( t \right) = at^{(n - 1)} e^{ - 2\pi bt} \cos (2\pi f_{0} t + \varphi ),(t > 0)$$
  where b is the filter bandwidth and n is the order of the filter.
3. After the filter filters, its power normalization (PN) process. Power normalized expression:
  9 $$U_{n} = \frac{{P_{n} (\omega_{g} )}}{\mu [\omega ]}$$
  where ${\text{Pow}}_{{\text{n}}} = {\text{U}}_{{\text{n}}}^{\vartheta }$ is the value processed by the Gammatone filter, and $\mu \left[ \omega \right]$ is the average power.
4. Power function nonlinear processing, its expression is ${\text{Pow}}_{{\text{n}}} = {\text{U}}_{{\text{n}}}^{\vartheta }$, $\vartheta$ is an exponential factor, and $0 < \vartheta$ < 1 is generally selected.
5. After the discrete cosine transform is performed on the nonlinearly processed signal, the EPNCC characteristic coefficient can be expressed as ${\text{y}}_{{\text{n}}}$.

After obtaining the EPNCC feature parameters through a series of processing, the EPNCC feature parameters are input into the convolution module for processing. The parameters of the convolution module refer to Table 1. The input network structure is shown in Fig. 1 below.

### Pulse time–frequency characteristic aliasing input

**Figure 2:**
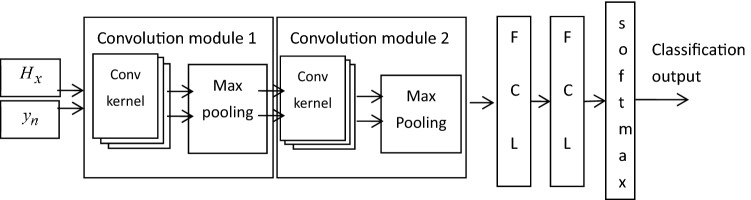
The overall structure diagram of pulse signal classification (Fully connected layer-FCL).

To fully characterize the pulse signal features, the frequency domain features and time-domain features are mixed into a convolutional neural network to identify and classify the mixed features. The general structure of the pulse signal classification network is shown in Fig. 2. The method first normalizes the periodic time-domain features and frequency-domain features and then inputs them into the convolutional network for training. Finally, the classification of the pulse signal is achieved by a softmax classifier. The classifier is suitable for the multi-classification requirements of this paper because it is computationally simple and does not require tightness and separation between classes. The number of categories corresponds to the number of clinical diagnostic categories to which the pulse belongs. Among them, CHF pulmonary edema is indicated by [1 0 0], respiratory failure is indicated by [0 1 0], and cardiogenic shock is indicated by [0 0 1].

The cost function used in this paper is cross-entropy, which is defined as:

10 $$C(\mathop {cl}\limits^{ \wedge } ,cl) = - cl\log (\mathop {cl}\limits^{ \wedge } ) - (1 - y)\log (1 - \mathop {cl}\limits^{ \wedge } )$$

When the cost function is reduced to a given acceptable error, this means that the network can be used for practical classification and recognition. For multilayer convolutional neural networks, the choice of the training algorithm is closely related to the computational complexity and classification accuracy of the network. Choosing an appropriate training rate algorithm can accelerate the convergence speed and reduce the occurrence of oscillations. The training method chosen in this paper is the momentum-driven stochastic gradient descent algorithm, which uses exponentially weighted averaging to make the computation of this gradient relevant to the previous method. As a result, the up and down oscillations of the gradient can be offset, thus accelerating the convergence rate. The specific structural parameters of the impulse classification network are shown in Table 3. In this paper, a total of 780 sets of data are acquired for network training and testing. Of these, 70% are used for network training and 30% are used to test the accuracy of the model.

**Table 3:** Network structure parameter table.

Classification network structure	Parameter configuration
Fully connected layer (output)	*k*
Activation function	Softmax
Cost function	Cross entropy
Network update algorithm	Stochastic gradient descent with momentum (sgdm)

## Experiments and results

### PPG data extraction and preprocessing

In this section, experimental analysis is performed to verify the effectiveness of the algorithm proposed in this paper. The pulse data were obtained from the MIT-BIH-mimic database provided by the Massachusetts Institute of Technology (MIT). The MIT-BIH database is one of the internationally recognized ECG databases that can be used as a standard and can effectively reflect the differences in pulse classification algorithms. In this experiment, the pulse signals of 39 patients were obtained from the simulated database^38^. The data are all ICU critical care patient pulse data from the MIMIC-||| database. These data were collected at a sampling frequency of 125 HZ. Among them, 39 groups of 1-min patient pulse data with clinical symptoms were classified into three categories: 16 groups of CHF pulmonary edema, 10 groups of respiratory failure, and 13 groups of centrally-derived shock. Twenty pulse signal samples were acquired for each group, and a total of 780 pulse data were used for network model construction. Among them, 546 were the training set and 234 were the test set.

**Figure 3:**
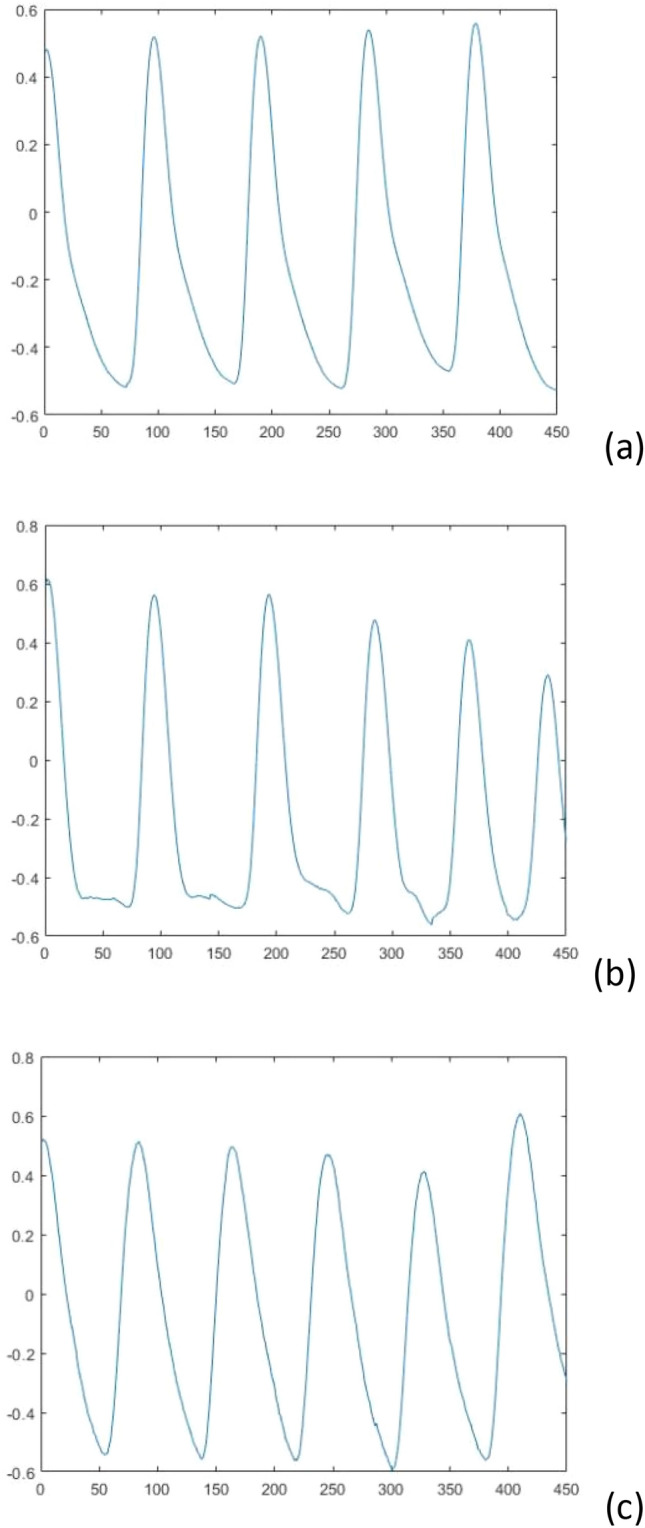
(**a**) CHFpulmonary edema; (**b**) Respiratory failure; (**c**) MIcardiogenic shock.

**Figure 4:**
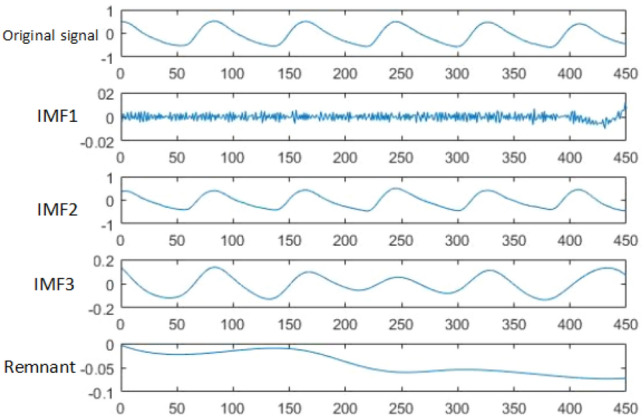
PPG signal EEMD decomposition.

In this experiment, MATLAB is used as the development platform to search the pulse data in the database for the peak points, and then group and segment the process. Since the sampling frequency of the pulse signal in the database is 125 Hz, 90 points were selected as a pulse cycle by observing the waveform, and five pulse cycles with a total of 450 data points were selected as the data samples for this experiment, starting from the peak. The sample plots of the pulse waveforms of the three clinical symptoms after segmentation are shown in Fig. 3 below. The EEMD decomposition of the pulse signal is shown in Fig. 4.

### Network simulation results and analysis

The network structure parameters are set as described in the previous chapter. After testing the network hyperparameters, the sgdm optimizer is selected to control the learning rate. The initial learning rate is 0.01, the learning rate reduction factor is 0.7, and the maximum iteration period is 50.

**Figure 5:**
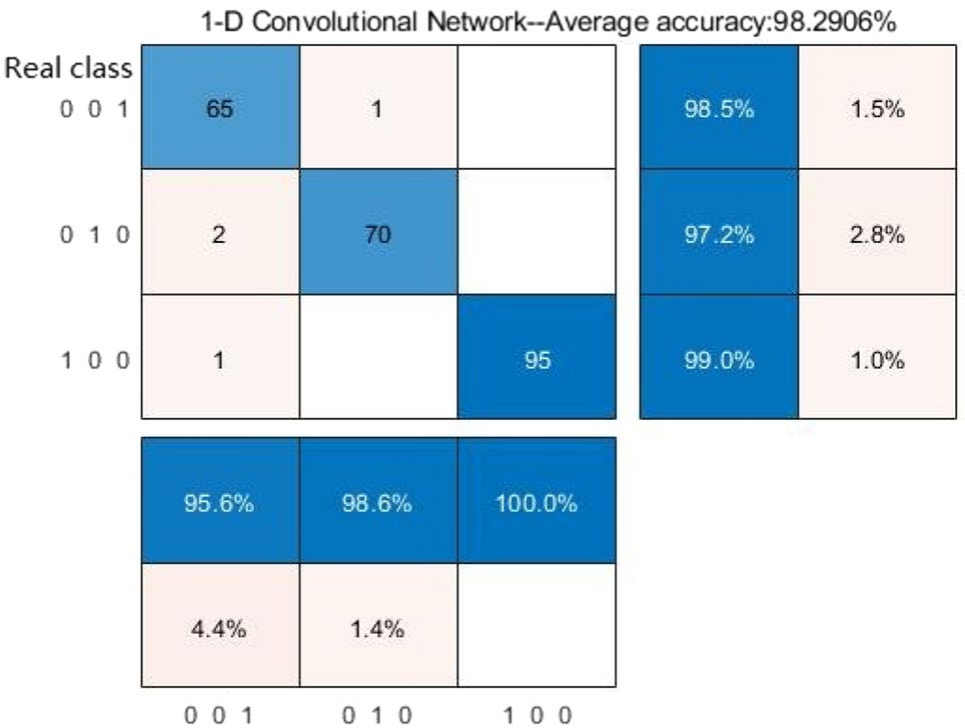
Multi-period, EPNCC feature classification confusion matrix.

The network is trained, classified, and validated by pulse period time-domain features (wavelet scattering) and the EPNCC coefficient conflation feature algorithm. The classification data in Table 4 are obtained through the confusion matrix in Fig. 5. The model obtained after training of the network was validated on the test dataset and the final classification accuracy was obtained as 98.29%.

**Table 4:** Pulse classification recognition rate of EPNCC-CNN.

Category	Recall rate (%)	Precision (%)
CHFpulmonary edema [1 0 0]	99.0	100
Respiratory failure [0 1 0]	97.2	98.6
MIcardiogenic shock [0 0 1]	98.5	95.6

To demonstrate the superiority of the method in pulse classification, pulse period feature training (sppft), pulse period (wavelet scattering) training (sppwsft), EPNCC feature training (EPNCCft), pulse period feature and EPNCC feature hybrid training (spp-EPNCCft), pulse period (wavelet scattering) and EMFCC hybrid features training (sppws-EMFCCft). They are all based on 546 sets of pulse data to train the model and then validated using 234 sets of pulse test samples.

By analyzing Tables 5, 6 and 7, we can get that the multi-period time-domain features of wavelet scattering processing have high accuracy for network identification and classification. Since the wavelet scattering processing can obtain multi-scale and multi-directional features with translation invariance and small deformation stability, this makes the pulse characteristics can be well characterized. When only EPNCC is selected as the network classification and recognition, its network classification is poor. Since the time domain features express most of the features of the pulse signal, EPNCC is used to express only a small part of the pulse that is ignored by the time domain features. The EMFCC method has also been experimented with within the paper, and the classification accuracy obtained by the EMFCC method was lower than that of the EPNCC method compared to the EPNCC method. This shows that the EPNCC method is more suitable for the pulse classification model in this paper.

**Table 5:** Recall rate of each training category.

Category project	Sppft (%)	Sppwst (%)	EPNCCft (%)	spp-EPNCC (%)	sppws-EPNCC (%)	sppws-EMFCC (%)
CHFpulmonary edema [1 0 0]	95.8	96.9	93.8	96.9	99.0	94.8
Respiratory failure [0 1 0]	88.9	86.1	83.3	94.4	97.2	95.8
MIcardiogenic shock [0 0 1]	86.4	97.0	86.4	95.5	98.5	93.9

**Table 6:** Precision of each training category.

Category project	Sppft (%)	Sppwst (%)	EPNCCft (%)	spp-EPNCC (%)	sppws-EPNCC (%)	sppws-EMFCC (%)
CHFpulmonary edema [1 0 0]	92.0	93.9	83.3	95.9	100	96.8
Respiratory failure [0 1 0]	90.1	95.4	95.2	95.8	98.6	93.2
MIcardiogenic shock [0 0 1]	90.5	91.4	90.5	95.5	95.6	93.9

**Table 7:** F-measure of each training category.

Category project	Sppft (%)	Sppwst (%)	EPNCCft (%)	spp-EPNCC (%)	sppws-EPNCC (%)	sppws-EMFCC (%)
CHFpulmonary edema [1 0 0]	93.86	95.37	88.24	96.40	99.50	95.89
Respiratory failure [0 1 0]	89.49	90.51	88.85	95.09	97.89	94.48
MIcardiogenic shock [0 0 1]	88.40	94.12	88.60	95.50	97.03	93.90

When the EPNCC is used as a complement to the time-domain features and the time-domain features are fused with the frequency-domain features, it enables complete characterization of the pulse signal, thus improving all the metrics of the network classification. The f-measure data metrics can show that the method used in the paper is feasible and effective. Analyzing the three types of clinical data in the table, there are some differences in the metrics obtained from the three data classifications, but the overall deviation is not significant. This may be due to the different number of training sets for the three types of clinical data, resulting in relatively poor network learning for certain symptom data.

The analysis of Table 8 shows that when only time-domain features are selected for network classification and recognition, the classification accuracy is 91.0% and 93.6%, respectively. When only frequency domain features are used for network classification and recognition, the classification accuracy is 88.5%. When the time domain features and frequency domain features were mixed, the classification accuracies were 95.7% and 98.3%, respectively. Accuracy is calculated by dividing all correctly predicted samples by the total samples. The accuracy rate is an evaluation of the overall performance. From the data in the table, the method proposed in this paper is effective in improving the pulse classification and recognition rates.

**Table 8:** Accuracy of each training category.

Category project	Sppft (%)	Sppwst (%)	EPNCCft (%)	spp-EPNCC (%)	sppws-EPNCC (%)	Sppws-EMFCC (%)
Accuracy	91.0	93.6	88.5	95.7	98.3	94.87

In this paper, other signal decomposition methods have also been experimented and the combination of EPNCC was proved to be more suitable for this pulse recognition model by comparing with EEMD. Because of the modal mixing in the signal decomposition process of EMD, it is not used in this paper for experiments. The decomposition methods used for comparison experiments in this paper are VMD, CEEMD, and CEEMDAN, respectively. The data obtained from the experiments are shown in Table 9 below.

**Table 9:** Experimental comparison of each signal decomposition method combined with PNCC (R: recall rate, P: precision).

Category project	CEEMD-PNCC		CEEMDAN-PNCC		VMD-PNCC		EEMD-PNCC	
	R (%)	P (%)	R (%)	P (%)	R (%)	P (%)	R (%)	P (%)
CHFpulmonary edema [1 0 0]	83.3	74.8	84.4	93.1	83.3	80.0	93.8	83.3
Respiratory failure [0 1 0]	68.1	81.7	87.5	81.8	79.2	87.7	83.3	95.2
MIcardiogenic shock [0 0 1]	83.3	82.1	95.5	90.0	83.3	79.7	86.4	90.5
Accuracy rate	78.63		88.46		82.05		88.46	

It can be obtained from Table 9 that the EEMD method is more applicable to the pulse recognition model compared to CEEMD and VMD. CEEMDAN is improved based on EEMD, and the results obtained by both methods are consistent for the decomposition of the pulse signal used in the paper; EEMD is sufficient to meet the research requirements of this paper, so EEMD is chosen.

At present, due to the limited source of data sets, the research method in this paper is limited to the data sets used. However, at the same sampling frequency, the data points contained in each cycle are the same, except for the different shapes of the pulse signals. Therefore, the feature processing method proposed in this paper is theoretically applicable to the analysis of other pulse datasets.

According to the analysis of Table 10, it can be found that the signal situation in the pulse data used in the literature^23^ is the same as that of the pulse data used in this article. The pulse single-cycle data point is 81, which is the same as the 90 data points in this article. In terms of data input dimensions, the input dimensions of this paper are nearly half lower than those in the literature^23^. The final classification accuracy rate is nearly 8% higher than that in the literature^23^.

**Table 10:** Pulse classification performance comparison.

literature	Author	Method used	Accuracy (%)
^23^	Yangsheng HU	Continuous wavelet transform to extract PPG features	83.81
^24^	Reit Kavsao Lu	The first and second derivatives of PPG signal extract time-domain features, k-NN classifier	94.44
^36^	Guohua Liu	Pulse time-domain characteristics and wavelet MFCC characteristics are mixed (CNN)	93.7
Method of this article		Wavelet scattering, EPNCC, CNN (softmax)	98.3

The sampling rate of pulse data in the literature^24^ is the same as the database used in this article, and the data points contained in each cycle are the same, which can be compared. Literature^24^ uses traditional feature extraction methods such as the distance between peaks and peaks to effectively extract pulse waveform features from normal healthy people, but this feature extraction method is limited in normal pulse waveforms. Abnormal pulse waveforms are not visible at every peak. There may be a situation where a certain wave peak is not obvious, which greatly increases the difficulty of traditional feature extraction methods and it is easy to ignore a certain part of the feature. The advantage of this paper is that PPG signals are processed directly through convolutional neural networks, and the EPNCC method is used to make the pulse characteristics more complete. It can be seen from the classification results that the research method is more effective than the method in the literature^24^.

The pulse data used in the literature^36^ and the pulse data in this article are from the same database. It is mentioned in the literature that single-period, multi-period, and MFCC features are used for mixed feature training, and finally a classification accuracy of 93.7% is obtained. However, this article only uses multi-period pulses for experiments and found that by adjusting the network parameters, the classification accuracy can be achieved when single-period and multi-period are mixed. Multi-period pulse data already contains the characteristics of single-period pulse data, and the superposition of the two will make the data redundant and increase the computational complexity. In this paper, the improved PNCC based on EEMD is used as a supplement to the time-domain features, and the final classification accuracy is 4.6% higher than that in the literature^36^. The reason may lie in the processing method in this article. The pulse signal of each frequency band is obtained through EEMD, and then after the coherence degree is screened, the PNCC processing is performed. One part of the PNCC processing is power-law nonlinear processing, which can amplify the pulse data information after processing, making the characteristics more obvious.

## Conclusion

The purpose of this study is to design a method to rapidly extract complete pulse signal features and to classify and identify them to improve the efficiency and accuracy of clinical diagnosis. This paper features the improvement of the traditional PNCC method by replacing the Fourier transform process in PNCC with EEMD and using this method to obtain feature information that is difficult to express in the time domain.

The signal in each frequency band is decomposed by EEMD and then processed by PNCC so that the tiny features can be amplified to make the pulse features more obvious. It is used as a supplement to the time-domain features to obtain the complete PPG signal characteristics. In this paper, the PPG signals provided in the MIT-BIH database are used for experimental validation to classify and identify three clinical categories of pulse signals. A mixture of multi-period PPG signals and EPNCC features are trained by convolutional neural networks, and the final classification results are better. The EPNCC features are selected as a complement to the time-domain features to avoid the problem of increasing computational complexity due to feature redundancy.

Due to the limited information on clinical symptoms associated with pulse signals in the MIT-BIH database. Therefore, it is difficult to construct a more complete and feasible pulse diagnosis model at this stage. However, in the future, the network model obtained in this paper can be updated and validated through migration learning. The method proposed in this paper hopes to explore more possibilities of the pulse signal in clinical diagnosis so that the pulse signal can be fully utilized to promote the development of intelligent clinical diagnosis.
